# Psychological Resilience of Healthcare Professionals During COVID-19 Pandemic

**DOI:** 10.1177/0033294120965477

**Published:** 2020-10-13

**Authors:** Faruk Bozdağ, Naif Ergün

**Affiliations:** Guidance and Psychological Counseling, Department of Educational Sciences, Faculty of Education, Istanbul University-Cerrahpasa, Istanbul, Turkey; Department of Educational Sciences, Faculty of Letters, Mardin Artuklu University, Mardin, Turkey

**Keywords:** Pandemic, psychological resilience, positive and negative affects, life satisfaction, healthcare professionals

## Abstract

The COVID-19 pandemic as a public health issue has spread to the rest of the world. Although the wellbeing and emotional resilience of healthcare professionals are key components of continuing healthcare services during the COVID-19 pandemic, healthcare professionals have been observed in this period to experience serious psychological problems and to be at risk in terms of mental health. Therefore, this study aims to probe psychological resilience of healthcare workers. The findings of this study showed that in order to raise psychological resilience of healthcare professionals working during the COVID-19 pandemic their quality of sleep, positive emotions and life satisfaction need to be enhanced. Psychological resilience levels of healthcare workers in their later years were found to be higher. Doctors constitute the group with the lowest levels of psychological resilience among healthcare workers. The current study is considered to have contributed to the literature in this regard. Primary needs such as sleep which are determinants of quality of life, life satisfaction and psychological resilience should be met.

## Introduction

The COVID-19 pandemic, which emerged in the Chinese city of Wuhan in December 2019 and has since spread to the rest of the world, has been described as a public health issue causing international concerns. The COVID-19 disease has caused and still causes health problems in over 3.3 million people worldwide as of May 3, 2020 (World Health Organization, 2020). Healthcare professionals have been observed in this period to experience serious psychological problems and to be at risk in terms of mental health (Black Dog Institute, 2020; Inchausti et al., 2020; Lai et al., 2020). Defined as a global pandemic, COVID-19 can lead to stress, apprehension and anxiety. Mood management is required to avoid exacerbation of stress and anxiety (Australian Psychological Society, 2020). It is essential that potential psychosocial impact of COVID-19 on healthcare workers is investigated (Arden & Chilcot, 2020).

Healthcare workers constitute the most affected group of people in the fight against the COVID-19 virus. Among the common mental effects of the pandemic are anxiety, panic, depression, anger, confusion, ambivalence and financial stress. Healthcare workers were observed to experience similar problems during previous pandemics (Black Dog Institute, 2020). Depression, anxiety and posttraumatic stress disorder are the most common psychological disorders that were reported particularly in healthcare professionals during the 2003 SARS and 2014 Ebola virus pandemics (Dong & Bouey, 2020; Maunder et al., 2006; Tam et al., 2004).

Studies have also shown that healthcare professionals are considerably more worried about catching the infection during a pandemic (Chua et al., 2004). Exposure to COVID-19 patients raises anxiety and fear of virus infection. As a result, levels of stress, depression and anxiety rise in healthcare workers and they might become traumatized (McAlonan et al., 2007). According to Cullen et al. (2020), particularly those working in public health, primary care, emergency service and intensive care are at the risk of developing psychological symptoms.

Studies conducted in China have revealed that healthcare workers are exposed to work overload, isolation and discrimination, and therefore they experience exhaustion, fear, affective disorders and sleep problems (W. Li et al., 2020). In a study conducted with 1563 healthcare workers, more than half of the workers (50.7%) reported depression symptoms, 44.7% anxiety and 36.1% sleep disorder (Liu et al., 2020). In a similar study carried out in Singapore, healthcare professionals were reported to experience depression, stress, anxiety and posttraumatic stress disorder (Tan et al., 2020).

As the research studies cited above show, it is crucial that mental health of healthcare workers is protected during the COVID-19 pandemic. In this regard, numerous reports coming out of China stress the importance of protecting mental health of healthcare workers (Denis et al., 2020). Achieving a sustainable success in the provision of healthcare services depends on the morale and sound mental wellbeing of healthcare workers (Low & Wilder-Smith, 2005). In the pandemic period, psychological resilience in particular rises in prominence (G. Smith et al., 2020). The COVID-19 pandemic is considered a threat to psychological resilience (Wang et. al., 2020). According to the American Psychological Association (2020), it is particularly crucial to promote psychological resilience of healthcare professionals during the pandemic.

Individuals who may be exposed to numerous hardships as well as shocking, destructive and stressful incidents differ in their reactions and coping strategies. Some individuals react to stressful and traumatic situations by yielding to psychological disorders such as anxiety and depression while others recover from negative mental state in a short time and resume their normal lives. This power that people who recover and resume their lives possess is referred to as psychological resilience in positive psychology approach (Doğan, 2015). Studies point to optimistic perspectives whereby most people become stronger fighting the difficulties they face through psychological resilience (Polizzi & Lynn, 2020). Psychological resilience can be defined, in the broadest sense, the individual’s ability to withstand hardship (Jackson et al., 2007). Defined as adapting to changes caused by stressful events in a flexible way and recovering from negative emotional experiences (Tugade & Fredrickson, 2004), psychological resilience impacts on the illness process and the subsequent health (Naeem et al., 2020). Psychological resilience is reported to be related to symptoms of anxiety and depression in healthcare professionals (Foureur et al., 2013).

Previous studies have argued that psychological resilience needs to be investigated through a systems approach that utilizes a multilevel interaction process between the individual and the environment. Psychological resilience is an ecological phenomenon and therefore it ought to be developed through environmental interactions such as family, community and society. The spaces individuals occupy contain the risk of producing various problems. However, the possibility of engendering positive outcomes may rise as well. Creating positive environmental conditions is likely to eliminate the risks for the individual (Brown & Westaway, 2011; Greene, 2002). According to Fergus and Zimmerman (2005), it is essential that psychological resilience is approached with an ecological perspective. Such an approach should consider the impact of environmental factors, as well as individual factors, in reducing risk elements. Therefore, any investigation of psychological resilience of healthcare workers needs to consider both environmental and individual factors.

The wellbeing and emotional resilience of healthcare professionals are key components of continuing healthcare services during the COVID-19 pandemic, as stated by the National Center for PTSD (2020). Thus, it is critical to anticipate the stresses linked to this process and providing support to healthcare professionals. Tracking and assessing the wellbeing of healthcare workers is important in terms of ensuring their successful reintegration with their coworkers in case they get infected. At this point, both institutional supports and selfcare strategies come into play. Therefore, a holistic assessment confirms the need to research psychological resilience of healthcare workers both at individual and environmental level.

### Purpose of the study

In Turkey, the number of people infected by the COVID-19 virus is 122 392 as of 3 May 2020 (World Health Organization, 2020) and this number is growing each day. This increase naturally affects the quality of healthcare services. Psychological resilience of healthcare workers needs to be improved and sustained in order to maintain the quality of healthcare services. Resilient mental state of healthcare workers influences not only their professional lives but their social and personal lives as well. Although the importance of healthcare workers has become established in Turkey, occasionally certain negative incidents occur. Healthcare workers are from time to time psychologically traumatized as they are stigmatized and discriminated against by certain segments of the society. On the other hand, the positive impact of the support offered to healthcare workers cannot be overlooked either. During the pandemic, for instance, the society in Turkey has been clapping from balconies in show of its appreciation to healthcare workers. To extend the effect of this positive atmosphere and enhance psychological resilience of healthcare workers at environmental and personal level, the current study attempts to investigate the factors impacting on psychological resilience of healthcare workers.

A wide gap has been reported in the literature concerning psychological resilience practices during long-term pandemic periods (Buheji et al., 2020). A related search of the literature revealed only a single research study (Lin et al., 2020) examining psychological resilience of healthcare professional during the COVID-19 virus outbreak. Considering the knowledge gap in the literature and with a view to improving the effectiveness of psychological support to be provided to healthcare workers, this study aims to probe psychological resilience of healthcare workers. The ecological framework was utilized to determine the variables impacting on psychological resilience. Accordingly, among the probed individual variables are gender, age, having children or not, taking personal precautions against the risk of becoming infected with the COVID-19 virus, worry about transmitting the virus to family/relatives, quality of nutrition and sleep, positive-negative affective state and life satisfaction, while environmental variables include weekly workload, organizational measures against the risk of becoming infected with the COVID-19 virus, perceived social support (perceived support by family, friends and someone special) and perceived organizational support.

## Methods

### Participants

Data were collected online for four days between 6 and 10 April 2020. A total of 214 healthcare workers (120 (56.1%) women and 94 (43.9%) men), including 66 doctors (30.8%), 69 nurses (32.2%) and 79 (36.9%) other healthcare staff with an age range of 20–65 (*M* = 33.29, *SD* = 6.82) participated in this study. Working hours of the participants were between 7 and 96 hours in a week (*M* = 43.46, *SD* = 11.37). The participants came from different cities in Turkey. Most of them were married (191 participants, 89.3%) and had children (118 participants, 55.2%).

### Measurement tools

#### The brief resilience scale (BRS)

The scale was developed by B. Smith et al. (2008) to measure individual psychological resilience. It consists of six items (three questions reverse) measured on a 5-point scale (1 Never suitable and 5 Completely suitable). The total score range was between 6 and 30. Higher scores on the scale indicate a higher level of psychological resilience. The Turkish version of the scale was adapted by Doğan (2015). The adapted scale was highly sufficient in terms of CFA values (χ^2^/df (12.86/7) = 1,83, NFI = 0.99, CFI = 0.99, GFI = 0.99, SRMR = 0.03, RMSEA = 0.05) and internal consistency coefficient (*α* = .88). In this study, the internal consistency coefficient was found as .82.

#### Survey of perceived organizational support (SPOS) brief form

The original scale of SPOS consists of 36 items and was developed by Eisenberger et al. (1986). However, they later recommended using a shorter version of the scale consisting of 17 items (Eisenberger et al., 1986). The Turkish version of SPOS was adapted by Azaklı (2014). Indeed, first the longer version of the scale was adapted to Turkish with the adaptation of the shorter version coming afterwards. In the Turkish version of the brief scale, there were 16 items in a 6-point Likert type scale (1: Completely disagree and 6: Completely agree) with high internal reliability (*α* = .96). In this study, the shorter version of the scale was used. The internal reliability of the scale in this study was also excellent (*α* = .92).

#### Multidimensional scale of perceived social support (MSPSS)

MSPSS was developed by Zimet et al. (1988) and adapted to Turkish by Eker and Arkar (1995). The Turkish version scale consists of three subscales (Significant other, Family subscale and Friends) with 12 items, measured on a 7-point Likert type scale (1: Very strongly disagree, 7: Very strongly agree). Internal consistency coefficient of the Turkish version was found .88 for total, .87 for family subscale, .85 for friend subscale, and .91 for significant other. In this study, internal reliability was excellent (MSPSS total: .97, Family: .96, Friends: .95, and Significant other: .96)

#### Satisfaction with life scale (SWLS)

SWLS consists of five items measured on a 7-point Likert type scale (1: Strongly disagree, 7: Strongly agree). It was developed by Diener et al. (1985) and adapted to Turkish by Köker (1991). Higher scores on the scale indicate higher levels of life satisfaction. The test-retest reliability coefficient of the scale was found .85. In the current study, the Cronbach alpha value was excellent (*α* = .91).

#### Positive and negative affect schedule (PANAS)

PANAS is a self-report measurement tool and consists of 20 items (ten items measure positive and other ten items measure negative affect) measured on a 5-point Likert type scale (1: Very slightly or not at all, 5: extremely). It was developed by Watson et al. (1988) and adapted to Turkish by Gençöz (2000). Scores range from 10 to 50 for both sets of items. Higher scores of positive items indicate having a high positive affect and lower scores of negative items indicate a less negative affect. The internal consistency coefficient of the Turkish version was .83 for negative affect and .86 for positive affect. In this study, internal reliability was .87 for positive affect and .88 for negative affect.

#### Questionnaire

Eight questions were prepared by the researchers to assess the situation of healthcare professionals during the COVID-19 pandemic. These questions included quality of sleep and nutrition, the risk of being infected by the virus, worry about transmitting the virus to their relatives etc. The questions were measured by a 5-point Likert type scale. The questions are: “Do you think adequate precautions are taken against the risk of coronavirus transmission in your institution? (1: The precautions are very poor, 5: The precautions are extremely enough)”, “Do you take adequate precautions individually to protect yourself against coronavirus? (1: Never, 5: Extremely)”, “What is your risk of getting coronavirus in the unit you work in? (1: Not at all, 5: Extremely)”, “Have you ever worked with someone who has a coronavirus infection? (1: Never, 5: Extremely)”, “Are you worried about being infected due to the risk at your work? (1: Never, 5: Extremely)”, “Are you worried to transmit coronavirus to your family members/relatives/friends because of your job? (1: Never, 5: Extremely)”, “How do you evaluate your nutritional quality? (1: Pretty inadequate, 5: Quite enough)” and “How would you rate your sleep quality for the last few weeks? (1: Pretty inadequate, 5: Quite enough)”.

### Procedures and data analysis

The entire surveys were prepared online and the link was shared with anyone who could voluntarily participate in the study. The participants from around 20 cities across Turkey filled out the questionnaire. The participants were informed about the study aims and procedures of the research. No reward was offered for participating. No personally identifiable information was requested.

For the analysis of the study, Pearson’s correlation analysis and hierarchical linear regression analysis were used. Before conducting the analysis, the normality of the items and the scale were checked. It was seen that skewness and kurtosis value of most of the items were between −1 to +1 and some items’ skewness and kurtosis value were between −3 to +3. The data can be considered to be normally distributed (Kim, 2013; Kline, 2011).

Moreover, sample size, univariate and multivariate outliers, normality, linearity, homoscedasticity, multicollinearity and independence of errors assumptions were calculated for hierarchical linear regression (Hair et al., 2014). No outliers ​​were found in the data set and the sample size of 214 participants can be considered as sufficient in accordance with the criteria [n ≥ 50 + 8 m (the number of independent variables in m)] (Tabachnick and Fidell, 2012). The scatter plots of the residues were examined, and it was observed that the assumptions of normality, linearity and homoscedasticity were met. For multicollinearity, it was assumed that the correlation coefficient between variables is less than .80, VIF (Variance Inflation Factor) is less than 10 and TV (Tolerance Value) is greater than .10 (Field, 2009). Bivariate correlations between the variables are given in Table 1. The fact that VIF values ​​of independent variables were between 1.25 and 4.77 (just three measurements were higher than 3) and TVs were between .21 (just one measurement was lower than .3) and .80 showed that multicollinearity assumption was met. Finally, the Durbin-Watson value was calculated as 1.97 and the assumption of independence of errors was met (Field, 2009).

**Table 1.** Means, standard deviation, and intercorrelations between variables.

	*M*	*SD*	1	2	3	4	5	6	7	8	9	10	11	12	13	14	15	16	17	18	19
1. Psych. Resilience	18.43	3.31	1																		
2. Perceived Org. Sup.	45.99	16.91	.116	1																	
3. Life Satisfaction	19.25	7.38	.330**	.351**	1																
4. Positive Affect	27.80	7.57	.371**	.136*	.184**	1															
5. Negative Affect	27.69	8.07	−.298**	−.274**	−.235**	−.023	1														
6. Someone Significant	18.14	8.47	.196**	−.009	.456**	.158*	.031	1													
7. Family members	20.10	7.22	.263**	−.027	.472**	.213**	.002	.796**	1												
8. Friends	17.44	7.30	.264**	.156*	.422**	.185**	−.087	.661**	.768**	1											
9. Age	33.29	6.82	.161*	.010	.133	−.029	−.146*	−.046	−.070	−.087	1										
10. Working Hours	43.46	11.37	.086	−.102	−.054	.150*	.021	.065	.082	.092	−.206**	1									
11. Having Child	–	–	−.007	−.004	.131	.002	−.036	.049	−.046	−.134	.643**	−.155*	1								
12. Organization Precaution	2.71	1.10	.052	.368**	.194**	.100	−.300**	−.076	−.078	.056	.135*	−.122	.017	1							
13. Personal Precaution	3.90	0.79	.158*	.193**	.121	.190**	−.042	.037	.054	.073	.088	.019	.001	.412**	1						
14. Personal Feel Risk	3.98	0.99	−.142*	−.239**	−.158*	−.024	.362**	−.002	−.045	−.156*	.020	.095	.089	−.227**	−.051	1					
15. Working Exp. COVID	2.18	1.45	−.074	.003	−.107	.047	.131	−.023	−.030	−.014	−.116	.185**	−.091	.002	.077	.316**	1				
16. Worry Get Virus	4.16	1.02	−.240**	−.185**	−.184**	−.173*	.522**	−.035	−.117	−.183**	.036	−.004	.081	−.205**	−.067	.448**	.034	1			
17. Worry Transmit Virus	4.61	0.83	−.122	−.130	−.126	.054	.429**	.003	−.003	−.109	.017	−.017	.109	−.233**	−.009	.391**	.117	.465**	1		
18. Nutrition	3.47	1.01	.184**	.124	.254**	.156*	−.111	.225**	.194**	.314**	.128	−.132	−.028	.185**	.247**	−.116	−.144*	−.202**	−.163*	1	
19. Sleep	3.06	1.16	.384**	.281**	.297**	.181**	−.195**	.194**	.246**	.258**	.073	−.184**	−.136*	.194**	.180**	−.284**	−.184**	−.269**	−.190**	.510**	1

** p < .01, *p < .05, N = 214. . Organization Precaution: Organization that a professionals worked at takes precautions against the virus, Personal Precaution: Individuals take precautions against the virus, Personal Feel Risk: Feeling risk of getting coronavirus in the unit that individuals worked in, Working Exp. COVID: Having working experiences with COVID-19 patient, Worry Getting Virus: Feeling worry to get coronavirus, Worry Transmit Virus: Feeling worry to transmit the virus to family members/relatives/friends because of the professionals, Nutrition: level of nutrition quality, Sleep: level of sleeping quality.

## Results

Means and standard deviation intercorrelation between variables were calculated and shown in Table 1. Psychological resilience significantly and positively correlated with life satisfaction, positive affect, sub-scales of perceived social support, participants’ age, taking personal precautions against coronavirus, nutrition and quality of sleep, meaning that an increasing level of psychological resilience leads to a higher level of the variables and vice versa. However, psychological resilience significantly and negatively correlated with negative affect, personally feeling in risk because of being healthcare professional, and worrying about being infected by the virus, meaning that decreasing level of psychological resilience leads to a rising level of the variables and vice versa.

Before regression analysis, t test for psychological resilience of women and men, and one-way ANOVA for types of occupations (doctors vs nurses vs other healthcare professionals) were calculated. The result of t test showed that differences between psychological resilience of women (M = 17.94, SD = 3.62) and men (M = 19.05, SD = 2.75) were statistically significant t (214) = -2.47, p = .014. The level of psychological resilience of men was higher than that of women. Difference between types of occupations in terms of the psychological resilience level indicated that although there were differences between the level of psychological resilience among the types of healthcare workers, the model was not statistically significant F (2, 211) = 2.96, p = .054. However, Bonferroni test showed that the level of psychological resilience of doctors (M = 17.70, SD = 3.01) and other healthcare professionals (M = 19.03, SD = 3.22) statistically and significantly differs, p = .048. But there were no statistical differences between doctors and nurses (M = 18.45, SD = 3.58), and nurses and other healthcare professionals.

A high correlation between psychological resilience and other variables showed further analysis was warranted (see Table 1). In Table 2, the hierarchical regression model was calculated to see how psychological resilience was predicted in terms of demographic variables, questions related to COVID-19, and variables related to perceived support and personal feeling that were used in the study. In model 1, demographic variables were calculated and it was found that gender, age, the types of occupation (doctors, nurses and other healthcare professionals), and having a child/children significantly predicted psychological resilience. But, having children (β = -.24) and being a doctor (β = -.20) negatively predicted psychological resilience. Overall, model 1 significantly predicted and explained 12% of the variance in the psychological resilience of healthcare professionals. Model 2 showed that demographic variables and questions related to COVID-19 together significantly predicted and explained 31% of the variance in the psychological resilience of healthcare professionals. In model 2, age, occupation, worry about becoming infected by the virus and quality of sleep significantly predicted the psychological resilience of healthcare professionals. Finally, Model 3 showed that all variables shown in Table 2 significantly predicted the psychological resilience of healthcare professionals and explained 43% of the variance. In model 3, age and occupation (doctor), quality of sleep, positive and negative affect, and life satisfaction significantly predicted the psychological resilience of healthcare professionals.

**Table 2.** Hierarchical Regression Model for Psychological Resilience of Health Professionals.

	Model 1				Model 2				Model 3			
Variables	*B*	*SE*	β	*p*	*B*	*SE*	β	*p*	*B*	*SE*	β	*p*
Demographic Variables												
Gender	1.00	.46	.15	.028	.56	.43	.08	.198	.45	.42	.07	.290
Age	.16	.05	.32	.001	.13	.04	.26	.003	.11	.04	.23	.004
Occupation^a^												
Doctor	−1.47	.54	−.20	.007	−1.37	.51	−.19	.007	−1.18	.48	−.16	.014
Nurse	−.34	.55	−.05	.536	.72	.53	.10	.173	.15	.50	.02	.759
Working Hours	.03	.02	.09	.221	.04	.05	.12	.064	.02	.02	.06	.305
Having Child	−.75	.28	−.24	.007	−.38	.26	−.12	.151	−.45	.25	−.15	.074
Questions Related COVID-19												essential
Organization Precaution					−.24	.21	−.08	.257	−.39	.21	−.13	.060
Personal Precaution					.24	.30	.06	.419	.27	.28	.06	.335
Personal Feel Risk					.09	.25	.03	.712	.06	.24	.02	.794
Work Experience with COVID-19 Patient					−.03	.15	−.01	.833	.03	.15	.01	.812
Worrying Getting Virus					−.63	.24	−.20	.009	−.11	.24	−.03	.649
Worrying Transmit Virus to Family Member					−.12	.29	−.03	.671	−.10	.27	−.02	.718
Nutrition					−.05	.24	−.02	.837	−.26	.24	−.08	.274
Sleep					1.13	.23	.40	.000	.87	.22	.31	.000
Perceived Support and Personal Feelings												
Positive Affect									.11	.03	.24	.000
Negative Affect									−.09	.03	−.22	.003
Someone Significant									−.01	.04	−.03	.764
Family Members									−.00	.06	−.01	.967
Friends									.05	.05	.10	.308
Life Satisfaction									.08	.03	.19	.014
Perceived Organizational Support									−.02	.01	−.11	.127
*R* ^2^	.119				.306				.427			
Adjusted *R*^2^	.093				.256				.364			
*R*^2^ Changed	.119				.187				.121			
*F* value	4.582				6.143				6.684			
*P* Value	.000				.000				.000			

a Reference : Other health professionals, *N* = 210, *p* < .05. . Organization Precaution: Organization that a professionals worked at takes precautions against the virus, Personal Precaution: Individuals take precautions against the virus, Personal Feel Risk: Feeling risk of getting coronavirus in the unit that individuals worked in, Working Exp. COVID: Having working experiences with COVID-19 patient, Worry Getting Virus: Feeling worry to get coronavirus, Worry Transmit Virus: Feeling worry to transmit the virus to family members/relatives/friends because of the professionals, Nutrition: level of nutrition quality, Sleep: level of sleeping quality.

## Discussion

Healthcare professional are forced to work under extremely difficult conditions owing to the COVID-19 virus outbreak (Greenberg et al., 2020). Under such circumstances, many essential healthcare workers become psychologically traumatized and need psychological support. It is argued that psychological supports to be offered to these workers ought to be based on psychological resilience models (Maunder et al., 2010). It is critical that psychological resilience of healthcare workers is protected and maintained during the pandemic (BC Centre for Disease Control, 2020; Santarone et al., 2020). This study too aimed to determine the factors impacting on psychological resilience with the hope of aiding psychological support services to be provided to healthcare workers.

Three models were tested through hierarchical regression analysis that was performed to specify the factors influencing psychological resilience of healthcare professionals. The first model looked into whether certain demographic variables predicted healthcare workers’ psychological resilience. The results showed that, in order of importance, age, having children, occupation and gender variables significantly predicted healthcare workers’ psychological resilience. Older age and being male heightened psychological resilience while being a doctor and having more children lowered psychological resilience. The second model revealed that, in order of importance, quality of sleep, age, worry about becoming infected by the virus and occupation variables significantly predicted healthcare workers’ psychological resilience. Thus, as the quality of sleep and age rose, so did healthcare workers’ psychological resilience whereas heightened worry about becoming infected by the virus and being a physician lowered psychological resilience level. The final model concluded that, in order of importance, the quality of sleep, positive affective state, age, negative affective state, life satisfaction and occupation significantly predicted psychological resilience of healthcare workers. Accordingly, higher levels of quality of sleep, positive affective state, age and life satisfaction raised the level of psychological resilience while higher negative affective state and being a doctor meant lower psychological resilience level.

According to the results of the last model, particularly the quality of sleep, positive emotional state, age and life satisfaction were found to have a crucial impact on improving psychological resilience of healthcare workers. It has been frequently noted in the literature that quality sleep acts as a protective factor against the psychological problems that healthcare workers might experience (Center for the Study of Traumatic Stress, 2020; Dewey et al., 2020; Inter-Agency Standing Committee, 2020; Lai et al., 2020; W. Li et al., 2020; Liu et al., 2020; Siyu et al., 2020). Healthcare workers face serious pressures that may cause psychological disorders, including anxiety, phobia, depression and insomnia (W. Li et al., 2020). According to Lai et al. (2020), a significant number of healthcare workers experience insomnia and develop symptoms of depression, anxiety and distress during the COVID-19 pandemic. In another study conducted with 1563 healthcare professionals, over half of them reported depression symptoms (50.7%), 44.7% anxiety and 36.1% insomnia (Liu et al., 2020). Similarly, a research study with 5393 participants showed that healthcare workers experienced depression, anxiety and insomnia (Siyu et al., 2020). Going without sleep for a long period of time is a risk factor for healthcare professionals (Inter-Agency Standing Committee, 2020). Therefore, it is crucial that healthcare workers’ basic needs such as food, fluids and sleep are met during quarantine time. Administrators of medical institutions need to ensure that healthcare workers get enough sleep (Dewey et al., 2020), thereby helping them stay psychologically more resilient. On the other hand, positive emotional state has been found to contribute to healthcare workers’ psychological resilience. Naeem et al. (2020) argue that individuals who actively develop positive emotions have higher psychological resilience. Positive emotions have been found to decline in the wake of COVID-19 pandemic (S. Li et al., 2020). Governments and particularly medical leaders can focus on changing people’s minds and thus heightening their psychological resilience levels (Buheji et al., 2020).

Busy work schedule and frequent exposure to negative incidents (deaths etc.) are considered as risk factors for healthcare workers. Healthcare workers at their later years, however, have been observed to manage this time better and to be psychologically more resilient. A positive relationship between age and psychological resilience indicates that healthcare workers cope better with crises as they get older. As they gain more experience, healthcare workers become more skilled at handling negative situations and grow psychologically more resilient. During the pandemic, one of the primary objectives should be taking necessary precautions to improve positive emotions and psychological resilience of healthcare workers. Research findings have shown that healthcare workers face mental health issues during the COVID-19 virus outbreak (Lai et al., 2020; W. Li et al., 2020; Liu et al., 2020; Siyu et al., 2020) and this naturally impacts on their life satisfaction. The S. Li et al. (2020) study has found an overall decline in life satisfaction following the COVID-19 pandemic. The present study also revealed that healthcare workers who are at risk and the most affected group by the pandemic grow more resilient as their life satisfaction rises. Accordingly, precautions ought to be taken to fight mostly commonly experienced problems such as anxiety, depression and apprehension in order to raise life satisfaction and thereby psychological resilience of healthcare workers.

Another result that came out of the current study is that negative affective state in healthcare workers significantly lowers their psychological resilience. Furthermore, doctors were found to have considerably lower psychological resilience levels compared to other healthcare workers. Individuals tend to develop negative emotions to protect themselves. People have reported heightened negative emotions during the COVID-19 virus outbreak. Prolonged negative affective state, however, may lead to various problems (S. Li et al., 2020). A negative relationship has been found between depression and anxiety, which are considered negative emotions in healthcare workers, and psychological resilience (Lin et al., 2020). This is consistent with the current study’s findings. It is possible to heighten healthcare professionals’ psychological resilience by lowering their negative emotions. However, this study’s finding that doctors have lower psychological resilience levels contradicts what Lin et al. (2020) found. In their study with 114 healthcare professionals, Lin et al. (2020) found that doctors’ psychological resilience is higher than other healthcare workers’. On the other hand, a study conducted in Singapore reported that during the 2003 SARS virus outbreak doctors carried more psychological symptoms risk compared to nurses (Chan & Huak, 2004), while another study revealed that frontline doctors in direct contact with patients developed even more serious symptoms of anxiety and depression (Siyu et al., 2020). These findings are consistent with the findings of the current study. Being in direct contact with patients, assuming more responsibilities and having a busy work schedule cause doctors to become exhausted and thus psychologically less resilient.

## Conclusion

The findings of this study revealed that in order to raise psychological resilience of healthcare professionals working during the COVID-19 pandemic their quality of sleep, positive emotions and life satisfaction need to be enhanced. Psychological resilience levels of healthcare workers in their later years were found to be higher. On the other hand, higher levels of negative emotional state lower psychological resilience level. Doctors constitute the group with the lowest levels of psychological resilience among healthcare workers. The research findings have revealed a significant portion of the variables impacting on the psychological resilience of healthcare workers in order that they could offer more quality service during the COVID-19 and similar pandemics. The current study is considered to have contributed to the literature in this regard. In addition, the result of the current study showed that quality of sleep, which is one of the primary needs, life satisfaction and positive-negative affairs are important prediction for the psychological resilience of healthcare professionals. Therefore, it can be indicated that for taking quality healthcare services and raise healthcare performance at work, primary needs such as sleep, and life satisfaction should be provided and healthcare professionals are to work in good conditions. The current study also concludes that in order to enhance positive emotions and weaken negative emotions of healthcare professionals, the workers’ needs ought to be prioritized in any practice.

## Limitations and recommendations

The comparatively small number of participants who provided data can be considered a limitation in terms of generalizability of the results. Future studies may reveal more generalizable results by collecting data from a higher number of healthcare professionals. Considering the current study is an example of cross-sectional research, it is necessary to conduct longitudinal studies that examine long-term effects of the pandemic. Positive and negative emotions were found to play a significant role in the model as the variables that predict psychological resilience of healthcare workers were analyzed. Therefore, further studies may have a better understanding of the issue through investigation of determinants of healthcare workers’ positive and negative emotions during the COVID-19 pandemic. In addition, life satisfaction and first needs such as sleep which can imply the quality of life were other important roles of impacting psychological resilience of healthcare professionals. Therefore, it can be worked relationships between healthcare workers’ quality of lives and psychological resilience during COVID-19.

## Author Biographies

**Faruk Bozdağ** works as a researcher at İstanbul University-Cerrahpaşa at Guidance and Psychological Counseling, Department of Educational Sciences, Faculty of Education, Istanbul University-Cerrahpasa, Istanbul, Turkey. His research interests include psychology of immigrants, cultural psychology, psychological counseling and school counseling.

**Naif Ergün** currently works as a researcher at the Department of Educational Sciences, Mardin Artuklu University, Turkey. His research interests include social psychology, addiction, school psychology and identity.
